# Identification and Expression Profiling of Chemosensory Genes in *Dendrolimus punctatus* Walker

**DOI:** 10.3389/fphys.2017.00471

**Published:** 2017-07-07

**Authors:** Su-fang Zhang, Hui-hui Liu, Xiang-bo Kong, Hong-bin Wang, Fu Liu, Zhen Zhang

**Affiliations:** Key Laboratory of Forest Protection, Research Institute of Forest Ecology, Environment and Protection, Chinese Academy of Forestry, State Forestry AdministrationBeijing, China

**Keywords:** development transcriptomes, olfactory genes, expression level, pheromone binding protein, pheromone receptor

## Abstract

*Dendrolimus punctatus* Walker is a serious pest affecting conifers in southern China. As extensive pesticide spraying is currently required to control *D. punctatus*, new control strategies are urgently needed. Chemosensory genes represent potential molecular targets for development of alternative pest control strategies, and the expression characteristics of these genes provide an indication of their function. To date, little information is available regarding chemosensory genes in *D. punctatus* or their expression profiles at different development stages and in various tissues. Here, we assembled and analyzed the transcriptomes of *D. punctatus* collected at different developmental stages and in a range of organs, using next-generation sequencing. A total of 171 putative chemosensory genes were identified, encoding 53 odorant binding proteins, 26 chemosensory proteins, 60 odorant receptors (OR), 12 gustatory receptors (GR), 18 ionotropic receptors (IR), and 2 sensory neuron membrane proteins (SNMPs). Expression analysis indicated that the antennae possess the largest number of highly expressed olfactory genes and that olfactory gene expression patterns in the eggs, larvae, and head were similar to one another, with each having moderate numbers of highly expressed olfactory genes. Fat body, ovary, midgut, and testis tissues also had similar olfactory gene expression patterns, including few highly expressed olfactory genes. Of particular note, we identified only two pheromone binding proteins and no pheromone receptors in *D. punctatus*, similar to our previous findings in *Dendrolimus houi* and *Dendrolimus kikuchii*, suggesting that insects of the *Dendrolimus* genus have different pheromone recognition characteristics to other Lepidopteran insects. Overall, this extensive expression profile analysis provides a clear map of *D. punctatus* chemosensory genes, and will facilitate functional studies and the development of new pest control methods in the future.

## Introduction

*Dendrolimus* spp. (Lepidoptera: Lasiocampidae) are among the most destructive defoliators of coniferous forests in China. Nearly 30 *Dendrolimus* species occur in China, of which *Dendrolimus punctatus* Walker is distributed widely in the south of the country. This pest primarily causes damage to *Pinus massoniana* Lamb.,one of the most important forest species in southern China (Chen, 1990). The outbreak pattern of *D. punctatus* is somewhat periodic; in an outbreak, a pine forest can be consumed in several days. Damaged pines appear burnt, leading to the effects of this pest being described as “fire without smoke.” The affected area previously extended to approximately 3,000,000 hectares, accounting for 50% of the total pest-damaged forest area in China (Chai, 1995). Hence, this pest has attracted the interest of many researchers of forest insects in China. Substantial information has been gathered on the biological characteristics of *D. punctatus*, including its population dynamics, natural enemies, the regularity of outbreaks, and management techniques for this pest (Chen, 1990; Zhang S.-F. et al., 2014). However, the outbreak mechanism of *D. punctatus* remains unclear and control methods that are continuously effective and efficient are still lacking. Annual heavy applications of pesticide are therefore an ongoing requirement for the control of *D. punctatus*, and the development of new control strategies is urgently required.Chemosensory genes are potential molecular targets for the development of alternative pest control strategies (Plettner, 2002). However, no information is currently available regarding the chemosensory genes of *D. punctatus*. Furthermore, chemosensory genes have important roles in the different developmental stages of insects (Liu et al., 2015; Wang et al., 2015). Both larvae and adults use their olfactory systems to detect chemical cues in the environment, for example, to search for food, mates, or adequate oviposition sites. Additionally, the expression patterns of various chemosensory genes in different organs may provide clues to their functions (Sun et al., 2012). Thus, it is necessary to identify *D. punctatus* chemosensory genes and examine their expression at different developmental stages and in different organs, as such information has potential to facilitate development of new pest control methods for this species.

Numerous studies have indicated that at least six gene families are involved in the detection of organic compounds in insects, including three receptor and two binding protein (odorant binding protein, OBPs, and chemosensory protein, CSPs) families, and the sensory neuron membrane proteins (SNMPs) (Su et al., 2009). The three receptor families expressed in insect olfactory sensory neurons include the odorant receptors (OR) (Touhara and Vosshall, 2009), ionotropic receptors (IR) (Benton et al., 2009), and gustatory receptors (GR) (Agnihotri et al., 2016). As these olfaction-related gene families typically contain many genes with extensive sequence diversity (Krieger et al., 2004; Robertson and Wanner, 2006; Engsontia et al., 2008; Tanaka et al., 2009), identifying them using purely homology-based methods may be inefficient. Earlier studies on the molecular details of moth olfaction have been restricted to insects with genomic data (Null, 2008; Tanaka et al., 2009), and the expense of genome sequencing has previously restricted the study of olfactory genes. Recently, however, the rapid progress of next-generation sequencing techniques has resulted in a number of sequencing studies of antennal transcriptomes that have identified olfactory-related genes in several moth species (Grosse-Wilde et al., 2011; Legeai et al., 2011; Bengtsson et al., 2012) and other insects (Mitchell et al., 2012; Andersson et al., 2013; Farias et al., 2015), demonstrating the power of transcriptomics for olfactory gene identification. Therefore, in this study, we assembled and analyzed developmental and organ-specific transcriptomes of *D. punctatus* using next-generation sequencing. This resulted in the identification of sets of putative OBPs, CSPs, SNMPs, ORs, GRs, and IRs, and characterization of their expression profiles in *D. punctatus*.

## Materials and methods

### Insect and sample collection

Pupae of *D. punctatus* were collected in Quanzhou County, Guilin city, Guangxi Province, China, in 2014. The pupae were reared in our research laboratory at 26 ± 2°C and 50 ± 10% relative humidity with a 16 h light: 8 h dark photoperiod. When they emerged as adults, they were sexed. Some insects were used immediately for tissue sample collection; male and female antennae and the head (without antennae) were removed and frozen in liquid nitrogen immediately. Some of the adults were reared continuously for the collection of tissue samples at various developmental stages. We collected egg (approximately 2–3 d), larvae (a mix of all larval instars), and pupae (approximately 5 d) samples and froze them immediately in liquid nitrogen. Fat body, midgut, testis, and ovary samples were collected from 5th instar larvae. For each tissue and developmental stage, samples were collected from more than eight insects; for small tissues (e.g., testis and ovary), samples were collected from more than 25 insects.

### RNA-Seq library preparation

Total RNA was extracted from each sample using TRIzol, according to the manufacturer's instructions (Invitrogen, Carlsbad, CA, USA). RNA degradation and contamination were monitored on 1.2% agarose gels. RNA purity and integrity were checked using a NanoPhotometer® spectrophotometer (IMPLEN, CA, USA) and an RNA Nano 6000 Assay Kit, with the Bioanalyzer 2100 system (Agilent Technologies, CA, USA), respectively. RNA concentration was measured using a Qubit® RNA Assay Kit in a Qubit® 2.0 Fluorometer (Life Technologies, CA, USA).

For each sample, 3 μg of total RNA was used for the synthesis of duplex-specific nuclease-normalized cDNA (Zhulidov et al., 2004; Bogdanova et al., 2008). All samples had RIN values >8. Sequencing libraries were generated using an Illumina TruSeq® RNA Sample Preparation Kit (Illumina, San Diego, CA, USA) following the manufacturer's recommendations, and four index codes were added to attribute sequences in each sample. To preferentially select cDNA fragments of 200 bp in length, library fragments were purified using the AMPure XP system (Beckman Coulter, Beverly, MA, USA). DNA fragments with ligated adaptor molecules on each end were selectively enriched using an Illumina PCR Primer Cocktail in a 10-cycle PCR reaction. Products were purified using the AMPure XP system and quantified using the Agilent high-sensitivity DNA assay on an Agilent Bioanalyzer 2100 system.

### Clustering, sequencing, *de novo* assembly, and assembly quality check

Index-coded samples were clustered on a cBot Cluster Generation System using a TruSeq PE Cluster Kit v3-cBot-HS (Illumina), according to the manufacturer's instructions. After cluster generation, library preparations were sequenced on an Illumina Hiseq 2500 platform (GnC Bio Company, Daejeon, Korea), generating 100-base pair (bp) paired-end reads. Raw sequence reads were exported in FASTQ format and deposited at the National Centre for Biotechnology Information (NCBI) short read archive under the BioProject accession number SRP095304. Next, we filtered adapters and deleted low-quality data from the raw reads, using Perl scripts developed in-house. Low-quality data, including reads containing >10% N (uncertain bases) and those where >50% of bases had sequencing error rates >1%, was removed to generate clean data. The Q20, Q30, GC-content, and sequence duplication level of the clean data were calculated simultaneously. All subsequent analyses were based on high-quality, clean data. Transcriptome assembly was accomplished using Trinity (vesion: trinityrnaseq_r20131110) (Grabherr et al., 2011), with min_kmer_cov set to minimum K-mer values derived using de Bruijn graphs. TGICL (TIGR Gene Indices clustering tools) (Pertea et al., 2003) was used for clustering of contigs to sequences without Ns and which could not be extended at either end, to obtain the final unigenes. Transcriptome assembly was assessed using benchmarking universal single-copy orthologs (BUSCO) to quantitate assembly completion, based on the percentage of sequences that aligned to highly conserved proteins (Simão et al., 2015).

### Annotation

Transcripts were annotated using the Trinotate pipeline (https://trinotate.github.io/). All assembled putative genes (henceforth, genes, for brevity) were searched with BLASTx against databases including Nr (NCBI non-redundant databases), Swissprot-Uniprot database, COG (Clusters of Orthologous Groups), GO (Gene Ontology), and KEGG (Kyoto Encyclopedia of Genes and Genomes) (E-value cut-off, 1e-5), and transcripts functionally annotated as the retrieving proteins or nucleic acid with highest sequence similarity. Then, GO classification was performed using Blast2GO (Conesa et al., 2005; Götz et al., 2008). Blast2GO annotation associates genes or transcripts with GO terms using hierarchical vocabularies. Genes are described in terms related to molecular function, biological process, or cellular component, allowing for meta-analyses of gene populations (Ashburner et al., 2000; Krieger et al., 2004)

ORs, IRs, GRs, OBPs, CSPs, and SNMPs were annotated in three steps: first, the chemosensory genes of other Lepidopteran insects, including *Heliothis virescens* (Vogel et al., 2010), *Spodoptera littoralis* (Legeai et al., 2011; Jacquin-Joly et al., 2012; Poivet et al., 2013), *Bombyx mori* (Gong et al., 2009), and two other *Dendrolimus* species (Zhang S. et al., 2014) were downloaded and candidate *D. punctatus* chemosensory genes were searched using the TBLASTN program against the local transcriptomes; second, identified genes were searched against the NCBI non-redundant protein sequences database and verified using TBLASTX; finally, for contigs with hits against genes of interest, open reading frames (ORFs) were identified and annotation verified by additional BLAST searches (http://blast.ncbi.nlm.nih.gov/Blast.cgi). Identified olfactory genes were submitted to the GenBank, and the accession numbers are listed in Table S1.

### Phylogenetic analysis

To further analyze the OR sequences, especially to find the candidate pheromone receptor candidates of *D. punctatus*, phylogenetic analysis of the predicted ORs protein sequences of *D. punctatus*, as well as their orthologs in two other *Dendrolimus* species (*Dendrolimus houi* and *Dendrolimus kikuchii*) and four other lepidopteran insects *Bombyx mori, Manduca sexta, Danaus plexippus*, and *Cydia pomonella*, were constructed.

We produced an alignment with the MAFFT using OR amino sequences of the up species. This alignment was used to produce a maximum likelihood phylogenetic tree using RAxML 8 with 1,000 bootstrap replicates (Stamatakis, 2006). Dendrograms were created and color labeled with FigTree software (http://tree.bio.ed.ac.uk/software/figtree/).

### Quantification of gene expression levels

The RESM program was used to calculated reads per kilobase of exon per million mapped reads (RPKM) expression values (Mortazavi et al., 2008; Li and Dewey, 2011), as this method considers the effect of sequencing depth and gene length for the read counts simultaneously, and is currently the most commonly used method for estimating gene expression levels from next-generation sequencing data. To minimize the influence of RNA output size differences among samples, we normalized the total reads by multiplying with normalization factors, as suggested by Robinson and Oshlack (Robinson and Oshlack, 2010). The RPKM of each gene was calculated based on the length of the gene and the number of reads that mapped to it. Hierarchical clustering using default options and the Euclidean distance similarity metric was performed on the normalized data.

### Quantitative real-time PCR (q-PCR)

Primers generating 100–200 bp products (Table S2) were designed from the annotated cDNA sequences, and verified by sequencing of the PCR products. Reverse transcriptase PCR using rTaq DNA polymerase (TaKaRa, Dalian, Liaoning, China) was performed for each primer pair before Q-PCR, to ensure that the correct products were amplified and no primer dimers were present. The standard curve method was used to measure relative mRNA expression levels normalized to reference genes. Five frequently used housekeeping genes (*beta-actin, GAPDH, 18S, tub*, and *EF-1A*) were used as reference genes in this experiment. Several chemosensory genes were selected as test genes; the selection criteria were that genes from different chemosensory groups should be included, along with those with relative high or low expression levels. The tested genes and reference genes were ligated into T-easy vectors (Promega, USA) and transferred into *Escherichia coli* DH5α competent cells (Tiangen, Beijing, China) for amplification. Plasmids were then extracted and 10-fold serial dilutions generated to construct Q-PCR standard curves to determine the PCR efficiencies of the primers for the target mRNAs and reference genes. All primers tested exhibited amplification efficiencies of 90–100%. Q-PCR was carried out using an ABI7500 (USA). The thermal cycling parameters were as follows: 2 min at 95°C, then 40 cycles of 20 s at 95°C, 20 s at 58°C, and 20 s at 72°C, followed by melting curve analysis from 58 to 95°C to evaluate the specificity of the PCR products. Three independent biological replicates (each biological replicate contained tissue from at least five insects) were performed for each tested item, and each reaction was performed in triplicate (technical replicates). We used GeNorm to determine which housekeeping gene was the most suitable (Vandesompele et al., 2002) among the five candidates (*beta-actin, GAPDH, 18S, tub*, and *EF-1A*), and the results indicated that all of them could be used as reference genes (*M* < 1.5). This may indicate that all samples tested in our experiments were collected under normal conditions, and that the insects were not subjected to stress; hence, the ubiquitously expressed house-keeping genes did not appear to be influenced by the conditions under which individual samples were collected. Thus, the expression values of chemosensory genes relative to Actin were used for comparisons with transcriptome data. The RPKM values of chemosensory genes from transcriptome data were also normalized to those of the *beta-actin* gene. Comparisons between Q-PCR and transcriptome data are presented in Figure S1.

## Results

### Identification of and phylogenetic analyses of chemosensory genes in *D. punctatus*

Using Illumina sequencing, we obtained *de novo D. punctatus* transcriptomes from different developmental stages and tissues. We totally obtained 38.6 Gb sequence data, and Trinity assembly of the sequencing data resulted in 163,354 contigs. BUSCOs were used to evaluate the completeness and accuracy of transcriptome assembly. Figure S2 shows the results of BUSCO matches, demonstrating 87.8% complete BUSCOs. Transcript comparisons were performed among the sequenced tissues and developmental stages (Figure S3) and the results showed that adults possessed the most unique transcripts among the different developmental stages (282 transcripts, Figure S3A), that the germ cells possess the most unique transcripts among different tissues (1255 transcripts, Figure S3B), and that the female antenna possesses more unique transcripts than the male antenna (Figure S3C).

Subsequently, we focused on olfactory-related genes, and identified a total of 53 OBPs, 26 CSPs, 60 ORs, 12 GRs, 18 IRs, and 2 SNMPs from the transcriptome data. These olfactory genes were subjected to further analyses as detailed below.

#### OBP genes

We totally identified 53 OBP genes in the *D. punctatus* transcriptome, this was a significantly larger number than those from the closely related species, *Dendrolimus houi* (*n* = 23) and *Dendrolimus kikuchii* (*n* = 27). This may be because we examined both development and tissue-specific transcriptomes in this study, whereas for *D. houi* and *D. kikuchii* only antenna transcriptomes were analyzed (Zhang S. et al., 2014). Of the identified *D. punctatus* OBP genes, there were three short OBPs and 50 full-length transcripts that could be divided into different groups according to their sequence properties: pheromone binding proteins (PBPs, 2 genes); general odorant binding proteins (GOBPs, 2 genes); classic OBPs (26 genes); plus-C OBPs (3 genes); and minus-C OBPs (17 genes).

**Figure 1:**
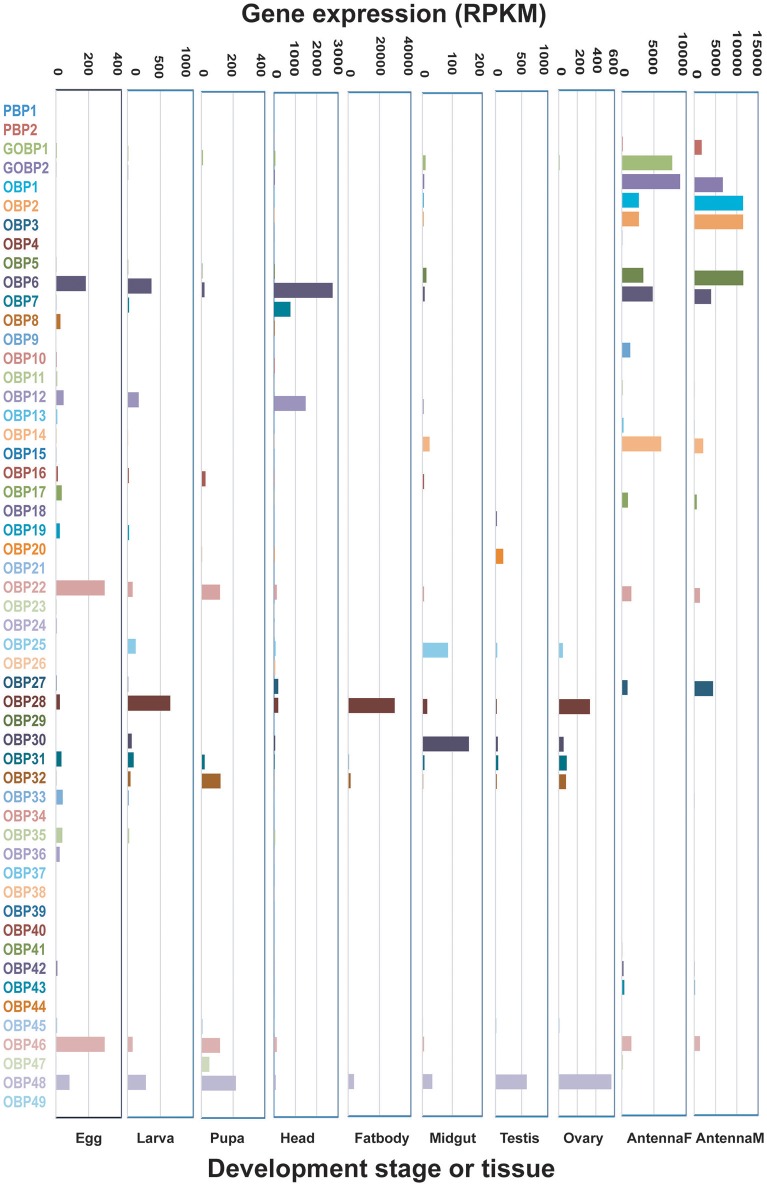
Expression patterns of candidate *Dendrolimus punctatus* odorant binding proteins (OBPs) at different developmental stages and in various organs. Transcript levels are expressed as reads per kilobase of exon per million mapped reads (RPKM).

OBPs with RPKM values ≥1000 were defined as highly-expressed genes, those with values 100–1,000 were defined as moderately-expressed genes, and those with values ≤100 were defined as weakly-expressed genes. Their expression levels in antennae were much higher than those in other adult tissues or at other developmental stages (Figure 1). For example, 10 highly-expressed OBPs were found in both male and female antennae, while the majority of other developmental stages and tissues had no highly-expressed OBP genes, with the exception of the head and fat body, which had two each. The antennae also had the most moderately-expressed OBP genes. OBPs exhibited the lowest expression in the midgut, testis, and ovary, where the majority of these genes exhibited weak expression. Egg, larva, and pupa had similar expression patterns, with three, four, and four moderately-expressed genes, respectively; other OBPs were expressed at low levels at these stages. The fat body and midgut tissues had the highest level of unexpressed OBP genes (*n* = 22 each) (Figure 1, Table 1). We selected several genes for verification of expression levels using Q-PCR (Figure S1); the results confirmed the reliability of the transcriptomic expression data.

**Table 1:** Numbers of olfactory genes at different expression levels at different developmental stages and in various organs of *Dendrolimus punctatus* Walker.

**Gene cluster**	**Expression level (RPKM)**	**Egg**	**Larva**	**Pupa**	**Head**	**Fat body**	**Midgut**	**Testis**	**Ovary**	**Antenna (F)**	**Antenna (M)**
	No expression	3	10	9	12	22	22	5	11	3	8
	Low (≤100)	46	38	48	34	26	29	45	40	31	28
OBP	Medium (100–1,000)	3	4	4	4	2	1	2	1	8	6
	High (≥1,000)				2	2				10	10
	No expression	1	3	4	2	8	6	1	2	2	3
	Low (≤100)	14	11	16	14	13	16	21	19	14	12
CSP	Medium (100–1,000)	8	8	3	6	1	1	1	2	5	5
	High (≥1,000)		1		1	1				2	3
	No expression	26	43	23	46	42	43	7	24	3	6
	Low (≤10)	33	17	36	14	18	17	51	36	24	36
OR	Medium (10–100)	1		1				2		32	18
	High (≥100)									1	2
	No expression	6	12	5	14	11	15	1	4		2
	Low (≤10)	10	4	11	2	5	1	14	12	8	8
IR	Medium (10–100)							1		7	5
	High (≥100)									1	1
	No expression	5	7	3	9	7	7		3	3	4
GR	Low (≤1)	5	3	6		2	2	3	6	3	3
	Medium (1–5)			1	1	1	1	6	1	3	3
	High (≥5)							1		1	
	No expression										
	Low (≤10)	2	2	1	2	2	2	1	2		
SNMP	Medium (10–100)			1				1			
	High (≥100)									2	2

*F, female; M, male; OBP, odorant binding proteins; CSP, chemosensory proteins; OR, olfactory receptors; IR, ionotropic receptors; GR, gustatory receptors; SNMP, sensory neuron membrane proteins. The numbers in the table indicate numbers of genes*.

#### CSP genes

**Figure 2:**
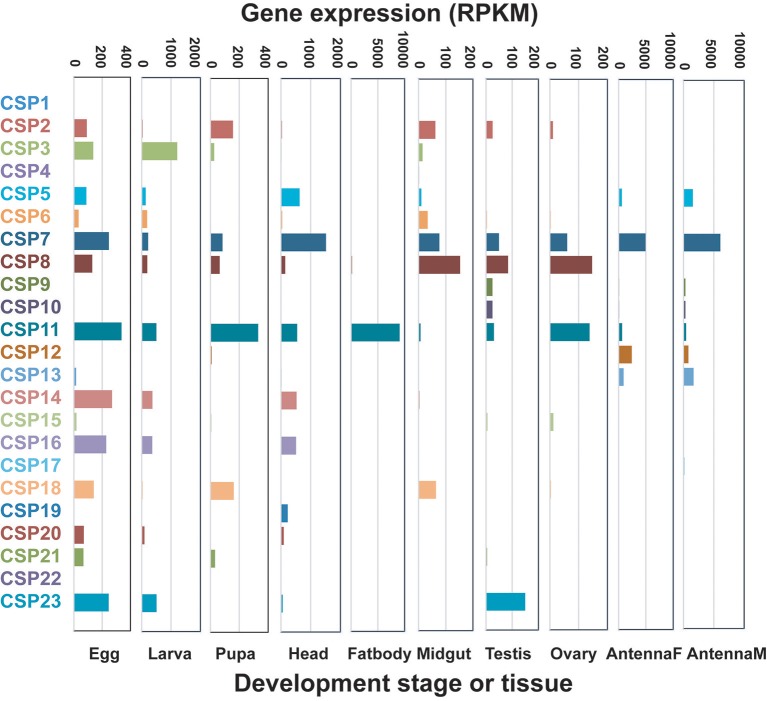
Expressions patterns of candidate *Dendrolimus punctatus* chemosensory proteins (CSPs) at different developmental stages and in various organs. Transcript levels are expressed as reads per kilobase of exon per million mapped reads (RPKM).

In *D. punctatus*, 26 CSP genes were identified that had relatively extensive and uniform expression patterns in different tissues and across developmental stages. Approximately eight CSPs reached moderate expression levels in female and male antenna, egg, larva, and head tissues. *CSP7* and *CSP11* showed relatively high expression levels in more than six tissues or developmental stages. Notably, *CSP11* exhibited the highest CSP expression levels in fat body tissue (Figure 2, Table 1). CSPs are believed to be multi-function proteins (Qiao et al., 2013), and their expression has previously been detected in fat body (Guo et al., 2016); however, the exceptionally high expression level of *D. punctatus CSP11* in fat body warrants further functional research.

#### OR genes

**Figure 3:**
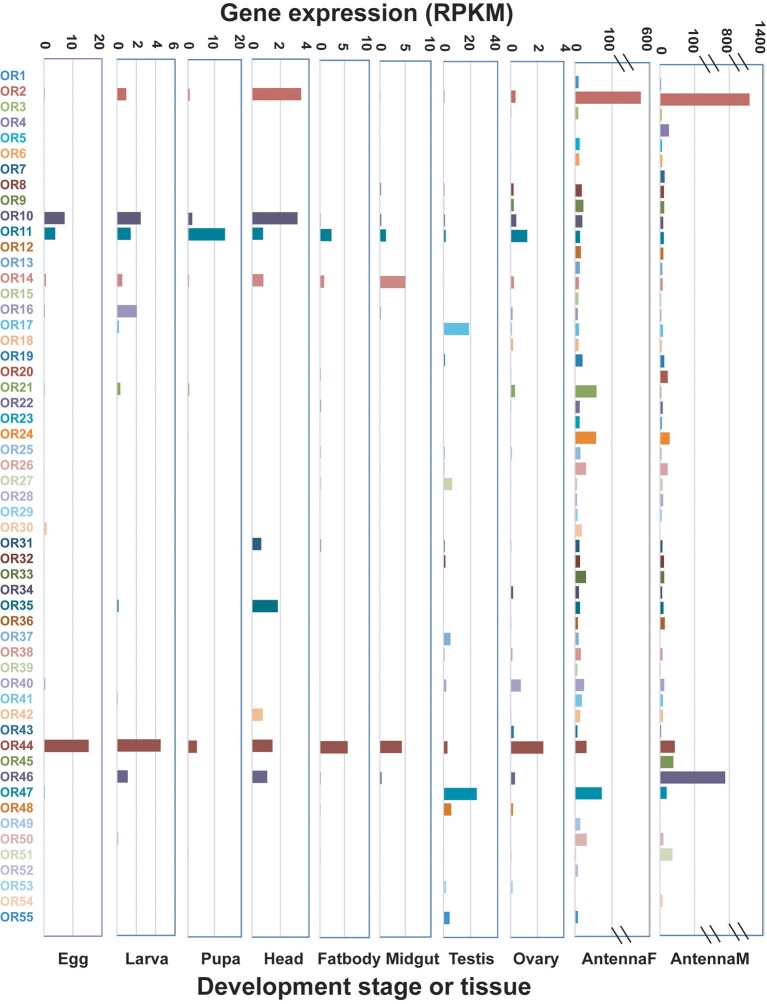
Expression patterns of candidate *Dendrolimus punctatus* olfactory receptors (ORs) at different developmental stages and in various organs. Transcript levels are expressed as reads per kilobase of exon per million mapped reads (RPKM).

In total, 60 OR genes were identified in *D. punctatus*. ORs with RPKM values ≥100 were defined as highly-expressed, those with values of 10–100 were defined as moderately-expressed, and genes with RKPM values ≤10 were defined as having low expression. OR expression levels in adult antennae were much higher than those in other tissues and developmental stages (Figure 3). In female and male antenna, 33 and 20 ORs were moderately- or highly-expressed, respectively, while <2 genes reached the threshold for moderate expression in each of the other developmental stages and organs (Table 1). Only two ORs were highly-expressed: olfactory co-receptor gene (*Orco*) (in both male and female antenna) and *OR46* (in male antenna only). The female and male antenna and testis had the fewest non-expressed genes (three, six, and seven, respectively), while more than 23 ORs were not expressed in other tissues or at other developmental stages. In larva and midgut, 43 ORs were not expressed (Table 1).

As no pheromone receptors (PR) were found in our previous work related to two other *Dendrolimus* species, *D. kikuchii* and *D. houi* (Zhang S. et al., 2014), phylogenetic analysis were performed to test whether some ORs from *D. punctatus* can be grouped as PR candidates. Comparisons were performed with OR genes from *D. punctatus*; four other species of Lepidoptera, *B. mori, Manduca sexta, Danaus plexippus*, and *Cydia pomonella* (Grosse-Wilde et al., 2011; Zhan et al., 2011; Bengtsson et al., 2012); and two other *Dendrolimus* species, *D. kikuchii* and *D. houi* (Figure S4). The *Orco* gene clustered with other lepidopteran *Orco* sequences, and high similarity was observed among the three *Dendrolimus* species; however, no ORs from *D. punctatus* were found among the lepidopteran pheromone receptor (PR) subfamily that occupied a separate branch.

#### GR, IR, and SNMP genes

Of the two other receptor classes (GRs and IRs), we identified a total of 12 GRs and 18 IRs in *D. punctatus*. Interestingly, the highest expression levels of GRs were in testis, rather than antenna (Figure S5, Table 1). Expression of GRs in the reproductive system has been reported in mammals (Wang, 2013); however, information about the expression and function of GRs in the testis of insects is sparse and further research is warranted. Eighteen IRs were identified, and these showed much higher expression levels in the antenna than in other tissues or developmental stages. Only sporadic IR expression was observed in testis and fat body (Figure S6, Table 1). Two SNMPs were identified that were expressed much more strongly in female and male antenna, consistent with their expression patterns in other species (Table 1).

Taken together, these results indicate that olfactory genes are differentially expressed across developmental stages and among tissues of *D. punctatus*.

#### Hierarchical clustering of *D. punctatus* chemosensory gene expression patterns

**Figure 4:**
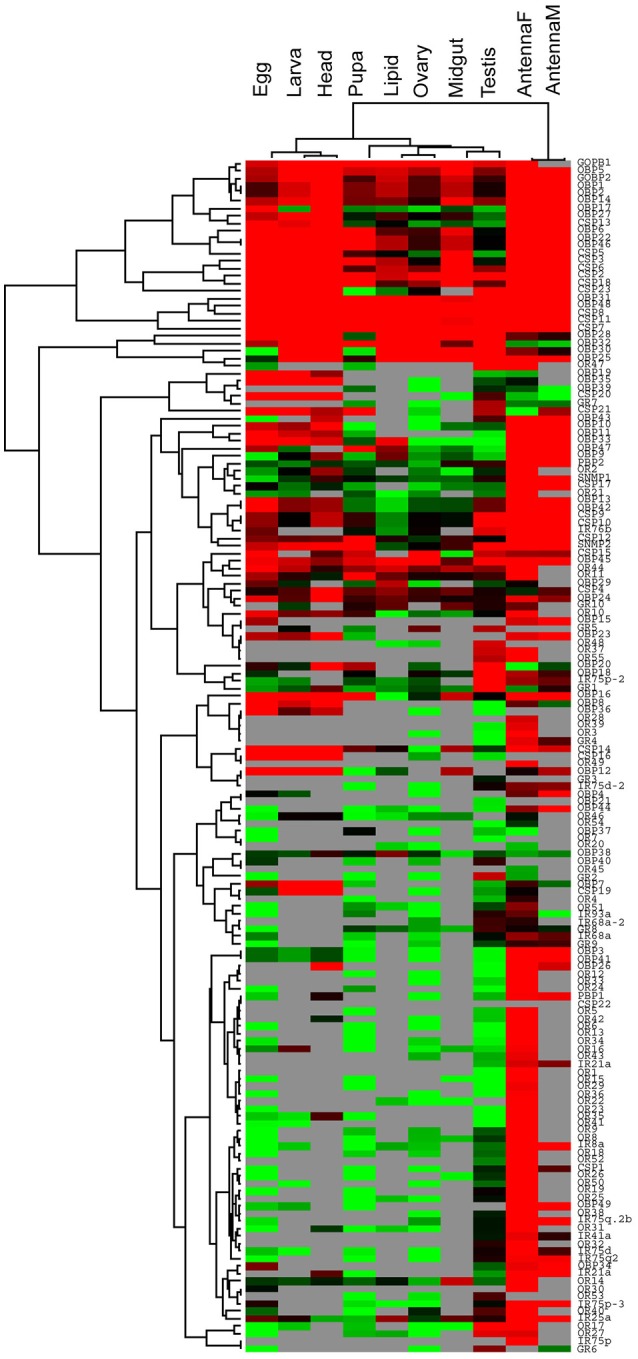
Hierarchical clustering of olfactory genes expressed at different developmental stages and in various organs of *D. punctatus*. Data from egg, larva, adult head, pupa, fat body, midgut, ovary, and testis tissues of fifth stage larvae, female antenna, and male antenna are included.

To examine the relationships between the olfactory gene expression patterns in the developmental stages and organs of *D. punctatus* tested, we performed hierarchical clustering of the expression data. Cluster analysis revealed that female and male antenna have similar olfactory gene expression patterns and that gene expression levels were relatively high in these tissues. Egg, larva, and adult head tissues had similar expression patterns and many genes were also highly expressed at these stages. Fat body, ovary, midgut, and testis tissues also exhibited similar expression patterns; however, few genes in these tissues were highly expressed. Likewise, few olfactory genes were highly expressed in pupal tissue (Figure 4).

## Discussion

In this study, we identified 53 OBPs, 26 CSPs, 60 ORs, 12 GRs, 18 IRs, and two SNMPs in the important pest, *D. punctatus*, using transcriptome data. These were larger numbers than those identified in *D. houi* and *D. kikuchii* in our previous studies (Zhang S. et al., 2014), where we identified 23 OBPs, 17 CSPs, two SNMPs, 33 ORs, and 10 IRs in *D. houi*, and 27 OBPs, 17 CSPs, two SNMPs, 33 ORs, and nine IRs in *D. kikuchii*. The differences in the number of genes identified is likely because we used transcriptome data from different developmental stages and organs in this study, whereas our studies of *D. houi* and *D. kikuchii* focused only antenna transcriptome data for identification of olfactory related genes.

In total, 53 OBPs were identified in *D. punctatus*; this is similar to the number identified in *B. mori* (44 OBPs), indicating that we obtained a comprehensive spectrum of OBP genes. However, as in *D. houi* and *D. kikuchii*, only two PBPs were found in *D. punctatus*. Therefore, it is possible that there are only two PBPs in the *Dendrolimus* genome, although additional genome sequence data will be required to confirm this hypothesis. The expression levels of OBPs displayed some interesting patterns in different tissues and across developmental stages. In the egg, all OBPs were weakly expressed, except *DpunOBP6, DpunOBP22*, and *DpunOBP46. DpunOBP6* also showed medium or high expression levels in larva, head, and antenna (both sexes); while *DpunOBP22* and *DpunOBP46* showed medium or high expression levels in pupa, head, and antenna (both sexes). Thus, these three genes may have important olfactory roles across all the developmental stages of *D. punctatus*. In larva, besides *DpunOBP6*, three other OBPs showed medium or high expression levels: *DpunOBP12, DpunOBP25*, and *DpunOBP48. DpunOBP12* was also highly expressed in *D. punctatus* head, but weakly expressed in other developmental stages and tissues. Blast comparisons demonstrated that *DpunOBP12* is a homolog of *SexiOBP11* (*Spodoptera exigua*), which is also strongly expressed in larval and head tissues (Liu et al., 2015). *DpunOBP25* was particularly highly expressed in *D. punctatus* larva, indicating that this gene may have a role in host localization during larval feeding, similar to *OBP2* in *S. exigua* (Liu et al., 2015). Interestingly, *DpunOBP48*, another OBP with relatively highly expression in larvae, was the most highly expressed gene in pupa, testis, and ovary, indicating that this gene may have multiple functions, similar to *OBP10* of *Helicoverpa armigera* and *Helicoverpa assulta* (Sun et al., 2012).

In *D. punctatus*, a total of 60 ORs were identified, significantly more than were found in *D. houi* and *D. kikuchii*, both of which were reported to harbor 33 ORs (Zhang S. et al., 2014). The number of ORs found in *D. punctatus* was comparable with those identified in other Lepidoptera species, such as *B. mori* (72), *M. sexta* (47), *Spodoptera littoralis* (47), and *Sesamia inferens* (39) (Zhang et al., 2013). Curiously, similar to *D. houi* and *D. kikuchii*, no PRs were detected among our data, although *OR1* was positioned very close to the PR cluster on phylogenetic analysis. While we previously assumed that PRs were not identified because we had not identified sufficient ORs overall in *D. houi* and *D. kikuchii*, these current data indicate that this may not be the explanation for their absence. We speculate that the pheromone identification genes of *Dendrolimus* may differ somewhat from those of other Lepidopteran insects, and that further functional analysis of the ORs is required to identify those acting as PRs in *Dendrolimus*.

The expression levels of ORs in different tissues and at developmental stages were compared. Given that approximately half of ORs exhibited moderate or high expression in antennae, while moderate expression of these genes was only observed sporadically in other tissues and developmental stages, expression levels of ORs were clearly higher in adult antennae than at other developmental stages or in other tissues. This is consistent with the function of ORs (Sakurai et al., 2004). *Orco* was highly expressed in both male and female antenna, consistent with the expression pattern of this gene in other insects (Benton et al., 2006; Zhang et al., 2016). Another gene, *DpunOR46*, was highly expressed, particularly in male antenna; this gene may be related to male-specific activities, such as sex pheromone recognition. Additionally, several genes were relatively highly expressed in egg (*OR44*), pupa (*OR11*), and testis (*OR17* and *OR47*). These genes may have dual functions, although further work is required for validation of this hypothesis. Notably, few ORs were expressed in the larval stage, and their expression levels were very low (Figure 3). In the larval stage, the insects may rely on olfaction to locate their food, and these weakly expressed ORs may also have roles in feeding; this hypothesis also requires further confirmation.

Another study compared the transcriptome profiles of *D. punctatus* egg, larval, pupal, and adult stages (Yang et al., 2016). This study also provided a list of olfactory genes, including 128 in total. Nine PBP genes were listed; however, only the first two were genuine PBPs, while the remaining seven were not OBPs. In addition, that study identified 18 ORs and, consistent with our results, no PRs were found. Of 15 GOBPs listed among their results, close inspection indicated that only two were GOBPs (*GOBP1* and *GOBP2*). Here, we identified 171 olfactory related genes, which was more than reported by Yang et al. This may be due to the antennal transcriptome analysis included in our study, which lead to the identification of more olfactory related genes.

Overall, we identified 171 chemosensory genes from *D. punctatus* transcriptomes of different developmental stages and tissues. In the antennae, a large number of highly-expressed olfactory genes were observed, consistent with their function; in egg, larva, and head tissue, a moderate number of highly expressed olfactory genes were identified; and in fat body, ovary, midgut, and testis few olfactory genes were highly expressed. Of particular note, we identified only two PBPs and no PRs, suggesting that *Dendrolimus* species may have different pheromone recognition characteristics compared with other Lepidopteran insects. The extensive expression profile analysis conducted in this study provides a clear map of *D. punctatus* chemosensory genes and will help to facilitate their functional characterization in the future.

## Author contributions

SZ designed and carried out sequence assembly and drafted the manuscript. HL carried out the laboratory experiments and assisted with sequence assembly. HW and XK collected the insects in the field. FL reared the insects in the lab. ZZ designed the experiments and modified the manuscript.

### Conflict of interest statement

The authors declare that the research was conducted in the absence of any commercial or financial relationships that could be construed as a potential conflict of interest. The reviewer AT and handling Editor declared their shared affiliation, and the handling Editor states that the process nevertheless met the standards of a fair and objective review.
