# Supplementary material for: Identification and Expression Profiling of Chemosensory Genes in Dendrolimus punctatus Walker
Source: Front Physiol. 2017 Jul 7;8:471. doi: 10.3389/fphys.2017.00471 (PMC5500615; doi:10.3389/fphys.2017.00471)
Supplement: Supplementary file 1 [file DataSheet1.docx]

Supplementary Material

Identification and Expression Profiling of Chemosensory Genes in *Dendrolimus punctatus* Walker

**Su-fang Zhang, Hui-hui Liu, Xiang-bo Kong, Hong-bin Wang, Fu Liu, and Zhen Zhang^*^**

*** Correspondence:** Dr. Zhen Zhang, [zhangzhen@caf.ac.cn](mailto:zhangzhen@caf.ac.cn)

**Figure S1.** Quantitative real time PCR (Q-PCR) validation of expression levels based on the reads per kilobase of exon per million mapped reads (RPKM) values generated using next generation sequencing. Q-PCR data are presented as means ± SD.

**Figure S2.** BUSCO matches for the *Dendrolimus* *punctatus* transcriptome assembly.

**Figure S3.** Venn diagram illustrating the transcript numbers expressed in different developmental stages and organs of *Dendrolimus* *punctatus*. **(A)** Comparison among different developmental stages, including egg, larva, pupa, and adult. **(B)** Comparison among different organs, including head, fat body, midgut, and germ cells (ovary and testis). **(C)** Comparison among sex-related tissues, including ovary, testis, female antenna (antenna-F), and male antenna (antenna-M).

**Figure S4. A maximum-likelihood dendrogram based on protein sequences of candidate odorant receptors (ORs).** ORs from *Dendrolimus punctatus* (Dpun), *Dendrolimus houi* (Dhou), *Dendrolimus kikuchii* (Dkik), *Bombyx mori* (Bmor), *Manduca sexta* (Msex), *Danaus plexippus* (Dple), and *Cydia pomonella* (Cpom) are included. *Orco* orthologs and sex pheromone receptors (PRs) are indicated.

**Figure S5.** Expression patterns of candidate *Dendrolimus punctatus* gustatory receptors (GRs) at different developmental stages and in various organs. Expression levels are expressed as reads per kilobase of exon per million mapped reads (RPKM).

**Figure S6.** Expression patterns of candidate *Dendrolimus punctatus* antenna ionotropic receptors (IRs) at different developmental stages and in various organs. Expression levels are expressed as

**Table S2 Primers used for Real-time PCR of selected genes**

| **Target/Primer Name** | **Sequence (5' to 3')** | **Gene accession number** |
| --- | --- | --- |
| *DpunGOBP1*-5′ | GCTGCGCTTCGAGAACG | KX585318 |
| *DpunGOBP1*-3′ | CCGCCTGGAGGATGAACT |  |
| *DpunOBP9*-5′ | GTATGCTTTGACGGAG | KX585275 |
| *DpunOBP9*-3′ | AAACTAAAGTAGCTCGGT |  |
| *DpunOBP5*-5′ | AACAAACGTGGACTTATCG | KX585271 |
| *DpunOBP5*-3′ | CATTACACCGTCAGACATCA |  |
| *DpunOBP45*-5′ | CAACACTCCATAGCCTCG | KX585311 |
| *DpunOBP45*-3′ | GCAGCCATAATCCACTCT |  |
| *DpunOBP31*-5′ | GCCTCCATAATCCGAAAG | KX585297 |
| *DpunOBP31*-3′ | TCCATCCAGCCACCCT |  |
| *DpunPBP2*-5′ | AAGAGTATGACAGCCAGTT | KX585317 |
| *DpunPBP2*-3′ | ATAATCCTCACGCCAAT |  |
| *DpunCSP1-5*′ | CATTCTGCTCGTTTGC | KX585412 |
| *DpunCSP1-3*′ | CACTTGATGTATGCCACT |  |
| *DpunCSP7-5*′ | GGTTTCATCGCTGTTTG | KX585418 |
| *DpunCSP7-3’* | CATTTCCCTTCCTCCAT |  |
| *DpunCSP9-5’* | TTTGATATTCAGCCACT | KX585420 |
| *DpunCSP9-3’* | TTATCGCTCTTACAACC |  |
| *DpunCSP14-5’* | TGGCATTAGCAGTCG | KX585425 |
| *DpunCSP14-3’* | ATCGGTGCATTCCTT |  |
| *DpunOR1-5’* | TGTTAGACAAAGGGAAGA | KX585320 |
| *DpunOR1-3’* | TGAAGTCATTGGCATTA |  |
| *DpunOR2-5’* | ATGCTCGTTACCATCAG | KX585321 |
| *DpunOR2-3’* | TTCCGTCAAGGTCTCA |  |
| *DpunOR4-5’* | TGGCTTTATGTCATCC | KX585323 |
| *DpunOR4-3’* | AATCGTGCTTGCTTAT |  |
| *DpunOR46-5’* | CACAAGCCCAAAGCC | KX585365 |
| *DpunOR46-3’* | CGGTAAGCGTATCAAGG |  |
| *DpunOR47-5’* | CAATAGTTATCGTCCTGC | KX585366 |
| *DpunOR47-3’* | ACCTCCTCGCTCTTCT |  |

Figure S1


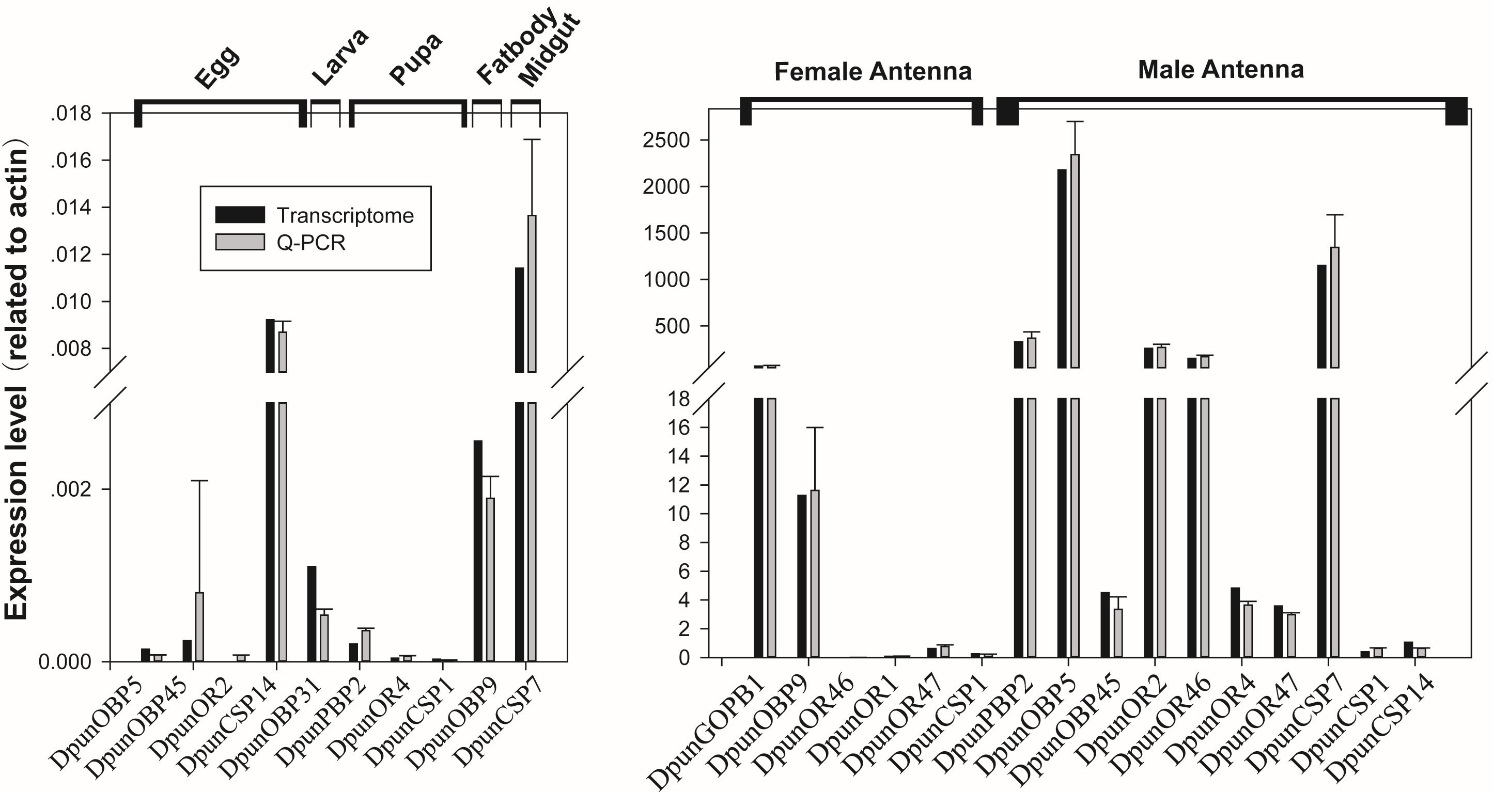


Figure S2


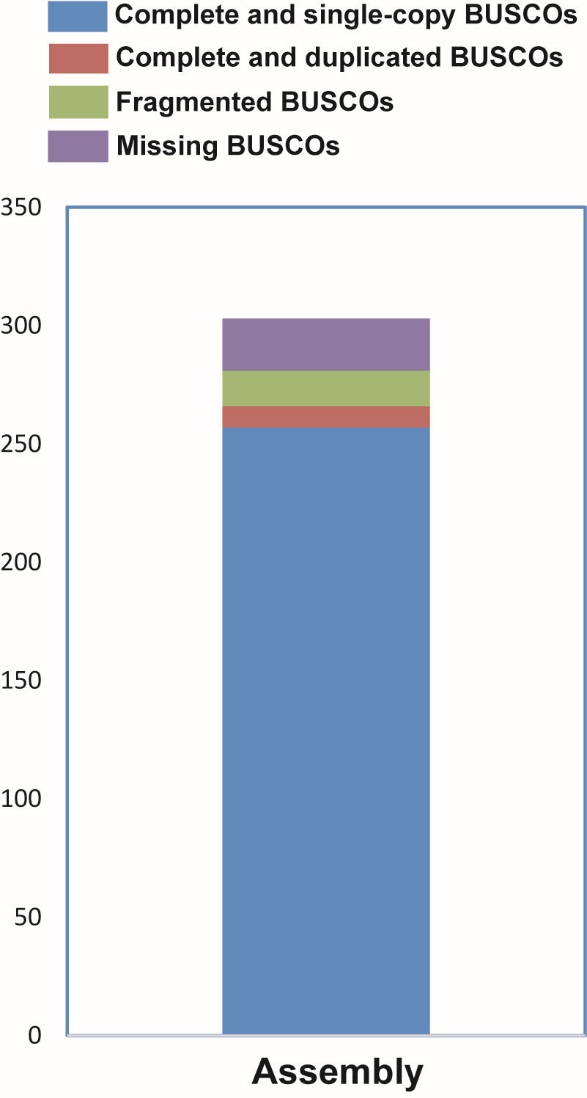


Figure S3


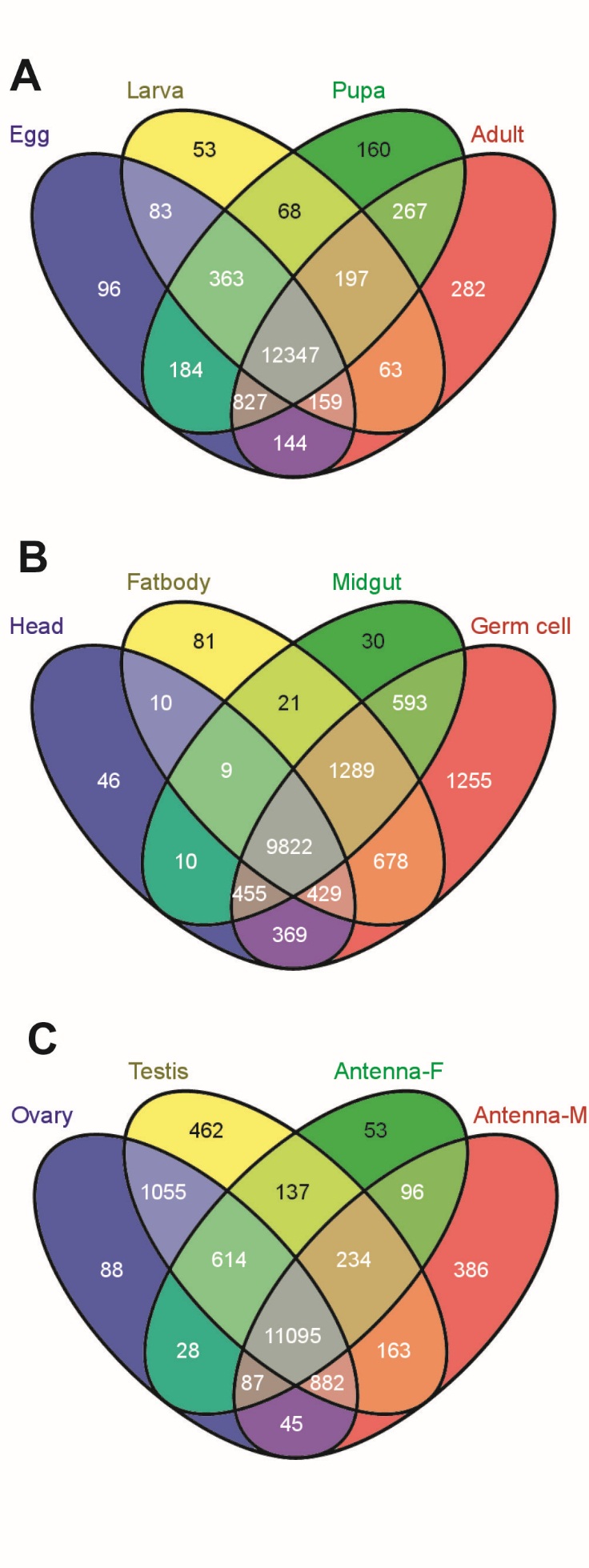


Figure S4


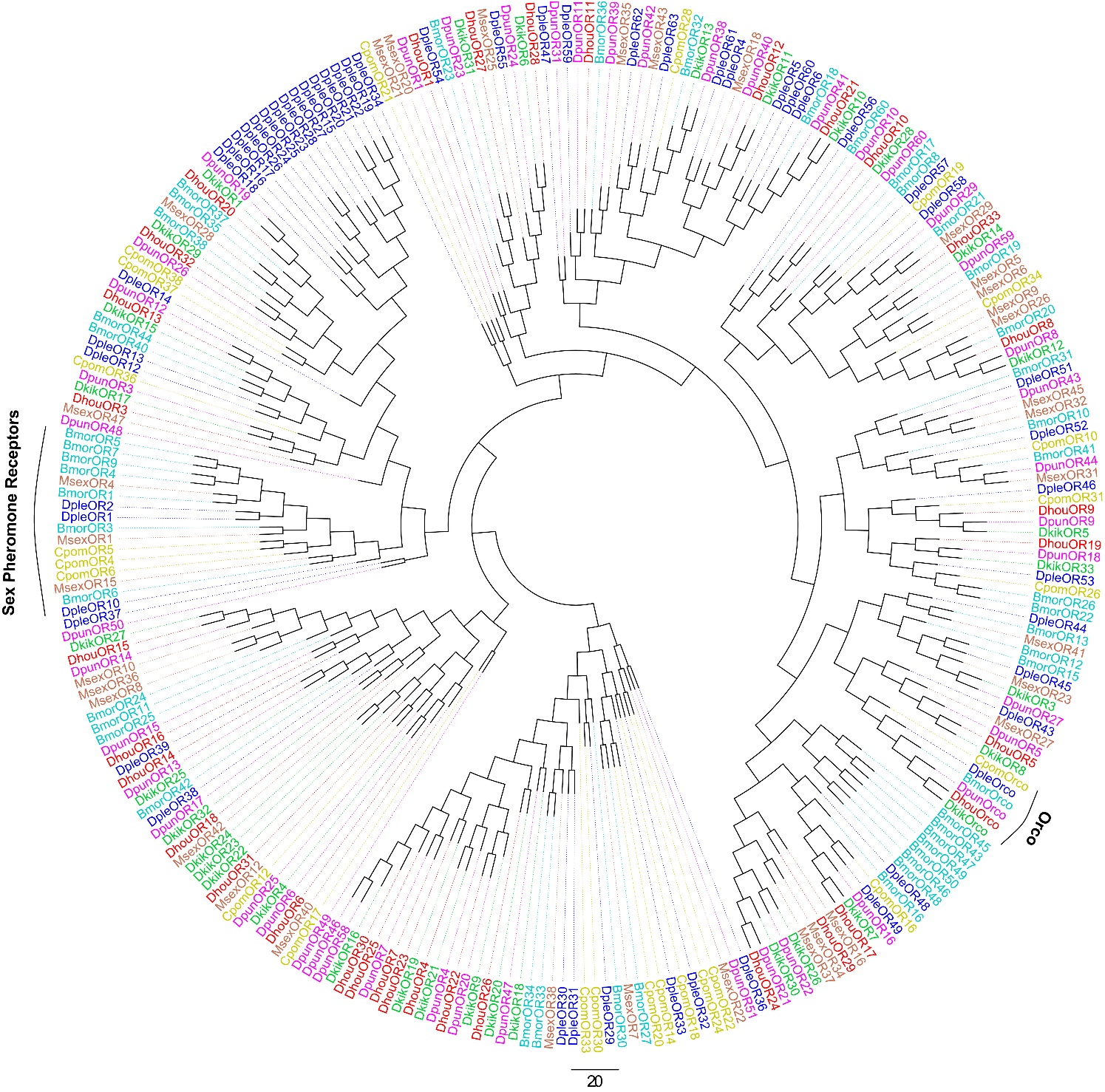


Figure S5


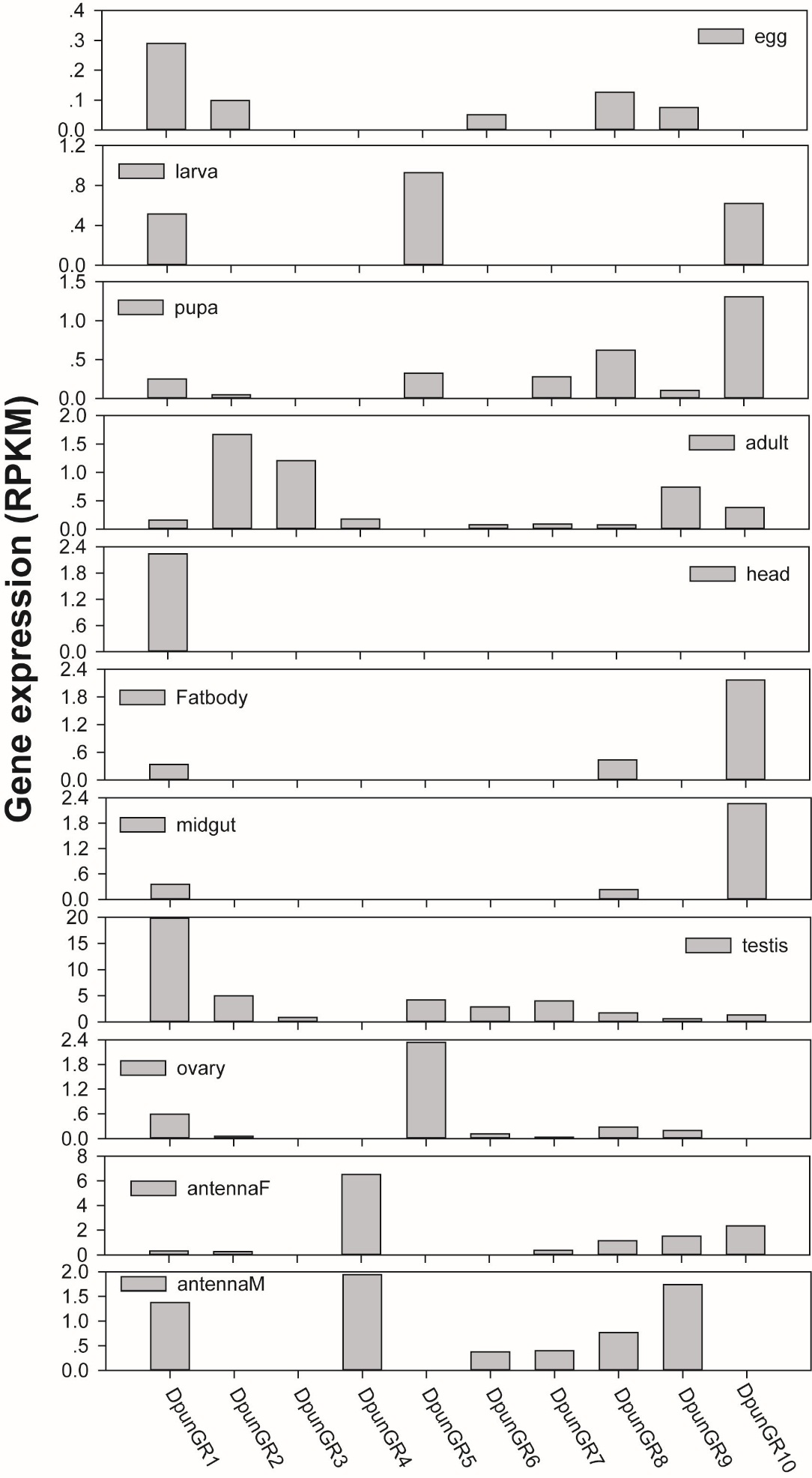


Figure S6


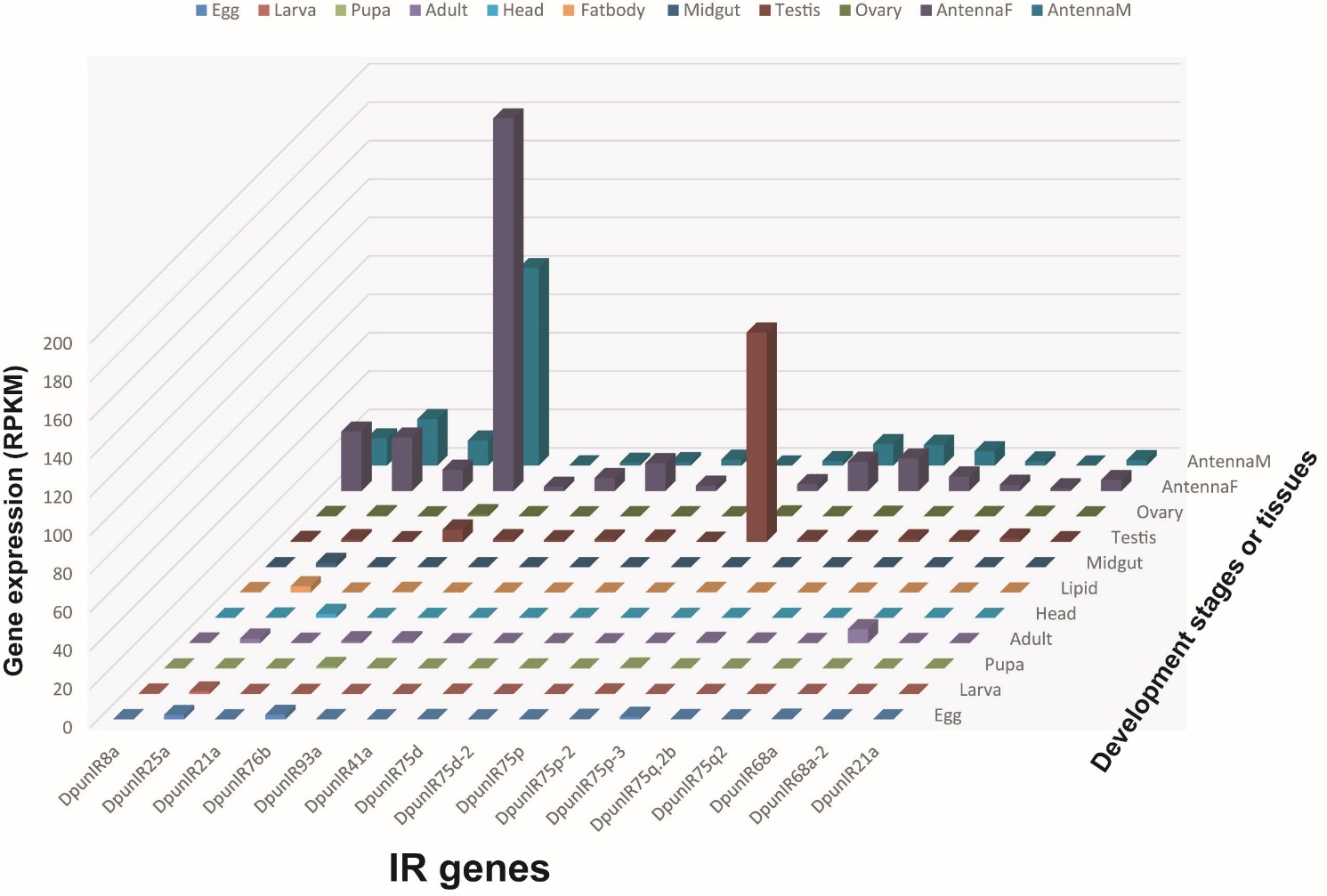


| Table S1. The NCBI Accession number of Identified sensory genes. | | | | | | | |  |  |
| --- | --- | --- | --- | --- | --- | --- | --- | --- | --- |
| **Gene ID** | **Accession** | **Gene ID** | **Accession** | **Gene ID** | **Accession** | **Gene ID** | **Accession** | **Gene ID** | **Accession** |
| DpunOBP1 | KX585267 | DpunOR1 | KX585320 | DpunGR1 | KX585380 | DpunIR8a | KX585392 | DpunCSP1 | KX585412 |
| DpunOBP2 | KX585268 | DpunOR2 | KX585321 | DpunGR2 | KX585381 | DpunIR25a | KX585393 | DpunCSP2 | KX585413 |
| DpunOBP3 | KX585269 | DpunOR3 | KX585322 | DpunGR3 | KX585382 | DpunIR21a | KX585394 | DpunCSP3 | KX585414 |
| DpunOBP4 | KX585270 | DpunOR4 | KX585323 | DpunGR4 | KX585383 | DpunIR76b | KX585395 | DpunCSP4 | KX585415 |
| DpunOBP5 | KX585271 | DpunOR5 | KX585324 | DpunGR5 | KX585384 | DpunIR93a | KX585396 | DpunCSP5 | KX585416 |
| DpunOBP6 | KX585272 | DpunOR6 | KX585325 | DpunGR6 | KX585385 | DpunIR41a | KX585397 | DpunCSP6 | KX585417 |
| DpunOBP7 | KX585273 | DpunOR7 | KX585326 | DpunGR7 | KX585386 | DpunIR75d | KX585398 | DpunCSP7 | KX585418 |
| DpunOBP8 | KX585274 | DpunOR8 | KX585327 | DpunGR8 | KX585387 | DpunIR75d-2 | KX585399 | DpunCSP8 | KX585419 |
| DpunOBP9 | KX585275 | DpunOR9 | KX585328 | DpunGR9 | KX585388 | DpunIR75p | KX585400 | DpunCSP9 | KX585420 |
| DpunOBP10 | KX585276 | DpunOR10 | KX585329 | DpunGR10 | KX585389 | DpunIR75p-2 | KX585401 | DpunCSP10 | KX585421 |
| DpunOBP11 | KX585277 | DpunOR11 | KX585330 | DpunGR11 | KX585390 | DpunIR75p-3 | KX585402 | DpunCSP11 | KX585422 |
| DpunOBP12 | KX585278 | DpunOR12 | KX585331 | DpunGR12 | KX585391 | DpunIR75q.2b | KX585403 | DpunCSP12 | KX585423 |
| DpunOBP13 | KX585279 | DpunOR13 | KX585332 |  |  | DpunIR75q2 | KX585404 | DpunCSP13 | KX585424 |
| DpunOBP14 | KX585280 | DpunOR14 | KX585333 |  |  | DpunIR68a | KX585405 | DpunCSP14 | KX585425 |
| DpunOBP15 | KX585281 | DpunOR15 | KX585334 |  |  | DpunIR68a-2 | KX585406 | DpunCSP15 | KX585426 |
| DpunOBP16 | KX585282 | DpunOR16 | KX585335 |  |  | DpunIR41a-2 | KX585407 | DpunCSP16 | KX585427 |
| DpunOBP17 | KX585283 | DpunOR17 | KX585336 |  |  | DpunIR76b | KX585408 | DpunCSP17 | KX585428 |
| DpunOBP18 | KX585284 | DpunOR18 | KX585337 |  |  | DpunIR21a-2 | KX585409 | DpunCSP18 | KX585429 |
| DpunOBP19 | KX585285 | DpunOR19 | KX585338 |  |  | DpunSNMP1 | KX585410 | DpunCSP19 | KX585430 |
| DpunOBP20 | KX585286 | DpunOR20 | KX585339 |  |  | DpunSNMP2 | KX585411 | DpunCSP20 | KX585431 |
| DpunOBP21 | KX585287 | DpunOR21 | KX585340 |  |  |  |  | DpunCSP21 | KX585432 |
| DpunOBP22 | KX585288 | DpunOR22 | KX585341 |  |  |  |  | DpunCSP22 | KX585433 |
| DpunOBP23 | KX585289 | DpunOR23 | KX585342 |  |  |  |  | DpunCSP23 | KX585434 |
| DpunOBP24 | KX585290 | DpunOR24 | KX585343 |  |  |  |  | DpunCSP24 | KX585435 |
| DpunOBP25 | KX585291 | DpunOR25 | KX585344 |  |  |  |  | DpunCSP25 | KX585436 |
| DpunOBP26 | KX585292 | DpunOR26 | KX585345 |  |  |  |  | DpunCSP26 | KX585437 |
| DpunOBP27 | KX585293 | DpunOR27 | KX585346 |  |  |  |  |  |  |
| DpunOBP28 | KX585294 | DpunOR28 | KX585347 |  |  |  |  |  |  |
| DpunOBP29 | KX585295 | DpunOR29 | KX585348 |  |  |  |  |  |  |
| DpunOBP30 | KX585296 | DpunOR30 | KX585349 |  |  |  |  |  |  |
| DpunOBP31 | KX585297 | DpunOR31 | KX585350 |  |  |  |  |  |  |
| DpunOBP32 | KX585298 | DpunOR32 | KX585351 |  |  |  |  |  |  |
| DpunOBP33 | KX585299 | DpunOR33 | KX585352 |  |  |  |  |  |  |
| DpunOBP34 | KX585300 | DpunOR34 | KX585353 |  |  |  |  |  |  |
| DpunOBP35 | KX585301 | DpunOR35 | KX585354 |  |  |  |  |  |  |
| DpunOBP36 | KX585302 | DpunOR36 | KX585355 |  |  |  |  |  |  |
| DpunOBP37 | KX585303 | DpunOR37 | KX585356 |  |  |  |  |  |  |
| DpunOBP38 | KX585304 | DpunOR38 | KX585357 |  |  |  |  |  |  |
| DpunOBP39 | KX585305 | DpunOR39 | KX585358 |  |  |  |  |  |  |
| DpunOBP40 | KX585306 | DpunOR40 | KX585359 |  |  |  |  |  |  |
| DpunOBP41 | KX585307 | DpunOR41 | KX585360 |  |  |  |  |  |  |
| DpunOBP42 | KX585308 | DpunOR42 | KX585361 |  |  |  |  |  |  |
| DpunOBP43 | KX585309 | DpunOR43 | KX585362 |  |  |  |  |  |  |
| DpunOBP44 | KX585310 | DpunOR44 | KX585363 |  |  |  |  |  |  |
| DpunOBP45 | KX585311 | DpunOR45 | KX585364 |  |  |  |  |  |  |
| DpunOBP46 | KX585312 | DpunOR46 | KX585365 |  |  |  |  |  |  |
| DpunOBP47 | KX585313 | DpunOR47 | KX585366 |  |  |  |  |  |  |
| DpunOBP48 | KX585314 | DpunOR48 | KX585367 |  |  |  |  |  |  |
| DpunOBP49 | KX585315 | DpunOR49 | KX585368 |  |  |  |  |  |  |
| DpunPBP1 | KX585316 | DpunOR50 | KX585369 |  |  |  |  |  |  |
| DpunPBP2 | KX585317 | DpunOR51 | KX585370 |  |  |  |  |  |  |
| DpunGOBP1 | KX585318 | DpunOR52 | KX585371 |  |  |  |  |  |  |
| DpunGOBP2 | KX585319 | DpunOR53 | KX585372 |  |  |  |  |  |  |
|  |  | DpunOR54 | KX585373 |  |  |  |  |  |  |
|  |  | DpunOR55 | KX585374 |  |  |  |  |  |  |
|  |  | DpunOR56 | KX585375 |  |  |  |  |  |  |
|  |  | DpunOR57 | KX585376 |  |  |  |  |  |  |
|  |  | DpunOR58 | KX585377 |  |  |  |  |  |  |
|  |  | DpunOR59 | KX585378 |  |  |  |  |  |  |
|  |  | DpunOR60 | KX585379 |  |  |  |  |  |  |
